# Evaluating Whether and How Public Health Event Information Frameworks Promote Pro-Environmental Behavior

**DOI:** 10.3390/ijerph20043721

**Published:** 2023-02-20

**Authors:** Lingyun Mi, Jiali Han, Ting Xu, Xuejiao Wang, Lijie Qiao, Tianwen Jia, Xiaoli Gan

**Affiliations:** 1 School of Economics and Management, China University of Mining and Technology, Xuzhou 221116, China; 2 School of Mechanics and Civil Engineering, China University of Mining and Technology, Xuzhou 221116, China

**Keywords:** public health emergency, coupling information, information framework, COVID-19 pandemic, pro-environmental behavior, control experiment

## Abstract

The major public health emergencies (PHEs) represented by the COVID-19 pandemic, while posing a serious threat to human health, have led people to rethink about the harmonious relationship between humans and nature. It is worthy to explore whether and how the framework effect of event information can be used to turn crises into opportunities to promote public pro-environmental behavior (PEB). Through a pre-and post-test control experiment, this study took the COVID-19 pandemic as a case, to explore the effects of four PHE information frameworks on promoting PEB, coupled with two information loss–gain frameworks and two information content frameworks. The results showed that all four information frameworks contribute to the public PEB. However, there are differences: only the environmental gain information effect is significant for PEB in the private sphere. The environmental loss and health gain information are effective for PEB in organizations. However, in the public sphere, all four information frameworks significantly motivate PEB. Further factorial analysis revealed that the interaction between the information content and loss–gain framework was not significant, with the latter playing the dominant role. These findings provide a new approach to how to develop the information framework effect and turn crises into opportunities to promote public PEB in the context of major PHEs.

## 1. Introduction

Public health and environmental sustainability are two of the major challenges facing the world in the 21st century [1]. In particular, the COVID-19 pandemic, which began in 2020, poses a serious threat to human health and also seriously disturbs normal production and life worldwide [2,3,4,5,6,7]. The COVID-19 pandemic is defined by the World Health Organization (WHO) as constituting a “public health emergency of international concern”. In the past three years, the variation and continuous spread of COVID-19 worldwide have produced a series of harmful and negative effects [8]. At the same time, more and more people have started to rethink how people and nature can live in harmony [9].

As the COVID-19 pandemic, a public health emergency (PHE), continues and changes, the way people work, travel habits, and consumption patterns have also changed significantly [10,11,12]. Several studies suggested that the COVID-19 pandemic event can be considered a major turning point, which can lead to the adoption of more sustainable lifestyles [13,14,15]. Some scholars are focusing on the positive impact of major PHEs, such as the COVID-19 pandemic event, on pro-environmental behavioral intentions [16,17,18]. However, Sun et al. discovered that people’s behavioral intentions do not always translate into actual behavior, and there is a gap between behavioral intentions and their actual behavior, by exploring and manipulating potential decision-making processes on the Internet of Things [19]. In terms of pro-environmental consumption, Grimmer and Miles found that there is also a gap between pro-environmental consumers’ intentions to purchase environmental products and their actual pro-environmental purchasing behavior [20], which is not conducive to the implementation of consumer PEB. How to bridge the gap between behavioral intentions and actual behavior in PEB becomes particularly important.

To promote PEB, there have been many successful studies on individual psychological factors, external contextual factors [21,22], and demographic characteristics. For individual psychological variables, scholars have mainly explored the predictive role of variables such as attitudes [23,24], values [25,26,27], subjective norms [28], environmental behavioral intentions [29], and perceived behavior control [30] on PEB. On external contextual variables, variables such as social norms [31,32], state policy [33], government incentives [34], and public media [32] have received attention. In terms of demographic variables, gender, age, and educational attainment have been widely used in the analysis of differences in PEB [35,36,37]. The existing research has focused on the influence of external contextual factors on PEB, but the role of information frameworks of major emergencies occurring in the external environment in motivating individuals to implement PEB has not received sufficient attention.

According to social information processing theory, when people are in a highly complex and uncertain environment, they will rely more on the various information provided by the social environment and constantly adjust their attitudes and behaviors to adapt to this uncertain and complex social environment [38]. The research conducted by found that people’s perceptions of information and sensitivity to the COVID-19 pandemic can influence their levels of knowledge, attitudes, perceived behavioral control, ultimate motivation generation, and provide the basis for promoting their PEB in society [39]. Broomell and Chapman found that people’s actual behavioral decision-making is more dependent on their perceptions, judgments, and feelings about the information they receive [40]. When people are confronted with emergencies in the external environment, external information can significantly influence individuals’ consciousness or behavior [41]. This shows that the framework effect of information dissemination can not only provide a powerful theoretical basis for increasing the persuasive power of information, but also create a new perspective for predicting individual behavioral decision-making. Therefore, whether and how to use the framework effect of information about PHEs to turn this crisis into an opportunity of promoting public PEB, bridging the gap between willingness to be pro-environmental and actual behaviors, becomes a potential approach to promoting public PEB.

Information frameworks refer to the different ways in which information represents choices, goals, and outcomes as related to behavioral decision-making [42]. Information recipients have different perceptions and judgments based on the information conveyed by information frameworks, which influence changes in their behavioral decision-making. The role of information frameworks on individual behavioral decision-making has received much research attention; for example, Mollen et al. found that the matching of information frameworks and norm types influenced consumers’ consumption of healthy food [43]. When positive framework information was used to express descriptive norms and negative framework information to express imperative norms, consumers were more likely to choose healthy food. Gallagher et al. used meta-analysis to explore the role of information frameworks on health communication behaviors [44]. They found that gain framework information was more persuasive than loss framework information for preventive behaviors such as preventing skin cancer, encouraging physical activity, and quitting smoking. Academics have now explored the important role of information framework in fields such as marketing, media studies, and medicine [45,46], but whether and how the information framework works to promote PEB in the context of the COVID-19 pandemic, a major PHE with global implications, needs to be validated. Therefore, to fill the above-mentioned research gap, this study divides the information frameworks into a gain framework that emphasizes the protection of the environment, and its positive consequences, as well as a loss framework that emphasizes the environmental damage and negative consequences, by taking the COVID-19 pandemic as a case. Additionally, it explores the effects of different information frameworks on public PEB decision-making through a pre-and post-test control experiment. Specifically, it examines whether two information loss-gain frameworks (emphasizing gains vs. losses) and two information content frameworks (emphasizing environmental vs. healthy outcomes) are conducive to promoting PEB.

This study extends previous work and contributes as follows: firstly, this study extends the study of promoting public PEB to the information management of PHEs, providing a new perspective on how to promote public PEB through information management in the context of major PHEs. Secondly, using the COVID-19 pandemic case, through a pre-and post-test experiment, we evaluated the effects of four coupled strategies of information content frameworks (environmental information and healthy information) and information loss–gain frameworks (a gain framework and a loss framework) on the public PEB decision-making, and then verified the effects of a single information strategy through factorial analysis, providing new insights into how to design information frameworks for PBE, which is an important addition to the existing literature on information frameworks. Finally, our study provides targeted recommendations on how policymakers can identify opportunities to promote public PEB through customized information design in major PHEs.

The rest of the paper is organized as follows: in Section 2, three sets of research hypotheses are presented through a review of the relevant theory and literature; Section 3 describes the methodology and process of the experimental study; Section 4 presents the results of the data analysis; Section 5 discusses the results obtained; and the final section explains the conclusions, practical implications, limitations of this research and future research perspectives.

## 2. Literature Review and Research Hypothesis

### 2.1. Information Content Framework

The information content is considered as an important factor in influencing PEB, but the effect of different information content varies [42]. A study by Abrahamse et al. found that promotional and educational information about environmental protection increased people’s knowledge of energy conservation, but did not lead to a substantial reduction in total household energy consumption [47]. In a study of group energy saving among university students, Mi et al. found that the experimental group with normative information + inter-group comparative feedback had the best energy savings (up to 24.23%) compared with the control group, by testing the effectiveness of four non-financial information strategies in promoting group energy savings [48]. Kamilaris et al. found that providing employees with specific information about the consequences of their environmental impact and targeted energy-saving methods was effective, which can promote employees’ energy-saving behavior [49]. This shows that the impact on the implementation of PEB varies significantly when different information is sent to people.

Most studies agree that information strategies have a positive effect on promoting PEB, but the effect of different information content frameworks is debated. In a study of household energy savings, Asensio and Delmas found that environment and health-based information treatments motivated 8% energy savings versus control and were particularly effective on families with children, who achieved up to 19% energy savings [50]. Additionally, Asensio and Delmas, in a home energy-saving experiment, found that a health-based framework (providing information on the impact of home electricity use on human health) resulted in more sustainable energy-saving behavior than a cost-based framework (providing information on the monetary cost of home electricity use) [51]. In a field experiment conducted in an Indian flat, Chen et al. found that providing environmental and health information was more effective in generating energy savings than providing monetary information [52]. Mi et al. found that environmental contribution feedback led to better energy savings than a combination of cost–benefit feedback interventions [53]. This shows that health and environmental information plays a greater role in promoting individuals’ environmental behavior than monetary information.

Some scholars have also focused on the impact of information frameworks on people’s health behaviors in the context of the COVID-19 pandemic. For example, a study by Corral-Verdugo et al. found that a positive environmental framework for the COVID-19 pandemic could buffer the negative effects of the COVID-19 pandemic, as well as mitigate people’s self-care and preventive measures against COVID-19 [54]. Si et al. studied people’s mask-saving intentions and behaviors during the pandemic, and they found that information campaigns had a positive impact on mask-saving intentions [55]. As the COVID-19 pandemic continues to evolve, human health and livelihoods have been greatly affected, leading to an increasing focus on the role of health and environmental information. However, there has been little research on the impact of COVID-19 pandemic healthy information and environmental information on public PEB, which may cause us to miss an important way to promote public PEB in the context of PHEs. Therefore, in this study, we focus our information content frameworks on environmental information and healthy information. Among them, the environmental information mainly refers to the ecological impact of the COVID-19 pandemic, and the health information refers to the public health impact of COVID-19 events.

Currently, the literature on which intervention, healthy information, or environmental information is more conducive to PEB is still relatively limited. In a study of energy consumption, using a sample of 120 UK household energy consumption data, Brandon and Lewis found that environmental information was more conducive to promoting household energy saving behavior than monetary information [56]. Castellari et al. found that health information was the main driver of willingness to pay, after conducting a study on the impact of health-related and environment-friendly related product information on the willingness to pay for functional foods [57]. There is also evidence that health-framed information may be more attractive than environmentally-framed information [58].

Therefore, to test which information content framework is more conducive to PEB in the context of the COVID-19 pandemic, health or environmental information, we propose hypothesis H1:

**H1.**  *The information content framework promotes public PEB decision-making, and the healthy information framework is more effective than environmental information.*

### 2.2. Information Loss–Gain Framework

In the fields of energy conservation and environmental protection, the application of the loss–gain framework has had an uplifting effect [59,60]. The framework assumptions of prospect theory state that the impact of the information presented on the behavioral decision is different, between the costs of engaging in risky behavior (i.e., the loss framework) or the benefits of avoiding risky behavior (i.e., the gain framework) [61,62]. When confronted with information about gains or benefits, people usually choose to avoid risk and reduce the loss of benefits, whereas when confronted with information about losses, they tend to choose to take risks and minimize losses. In our study, the gain framework was defined as the benefit to the environment or health resulting from the COVID-19 pandemic, and the loss framework as the negative impact to the environment or health resulting from the COVID-19 pandemic.

The loss–gain framework information has been found in studies on predicting disease [63], health management [64], and climate change [65]. In recent years, the impact of the information loss–gain framework on public emotions and behaviors has also received increasing academic attention. Nabi et al. used a meta-analysis to analyze the literature on the impact of emotions on the loss–gain framework in 25 studies, and they found that the information loss–gain framework directly influenced subjects’ emotional responses, with the gain framework inducing positive emotions and the loss framework inducing negative emotions [66]. Mays and Zhao studied the impact of loss–gain framework information on young adult women’s intention to indoor tanning (IT), and they found that loss–gain framework information reduced women’s IT behavioral intentions by increasing fear [67]. Ort et al. examined the effects of a single gain and loss framework vs. a combined gain–loss framework on sun protection intentions and sun protection behaviors, and they suggest that mixed-framework or pure loss framework information was more likely to increase health behavioral intentions than pure gain framework information [68]. However, in a neutral framework control condition, after examining the effects of air conditioning use among students in Japanese university dormitories, Iwasaki et al. found no significant difference between the energy use of students who received loss framework information or gain framework information [69].

Although studies have confirmed the positive effects of the information loss framework on promoting PEB, there is still debate on which is more effective, the information gain framework or the information loss framework. Through an inter-group experiment, Dai et al. found that for interdependent individuals, the gain framing message has more positive influence on the intention of green consumption behavior, whereas the loss framing can better promote independent individuals’ willingness [70]. Bager and Mundaca proved that loss framework information can result in a significant reduction (7–11%) in daily energy demand [71]. Ghesla et al. found in the study of a household electricity saving experiment that the loss of framework was able to reduce household electricity use by 5% compared with a control group [72]. Ropret and Knežević found that the loss framework was more effective in changing behavior and intentions in a study of the effect of framework effects on PEB decision-making [73]. However, Kim, S.-B. and Kim, D.-Y. [74] found that the gain framework was better at influencing the PEB (i.e., visit intentions and water and energy savings) of hotel guests compared with the loss framework.

Thus, it is valuable for us to further explore which information framework is more effective in promoting public PEB in the context of the COVID-19 pandemic. Though the loss framework information leads to stronger negative emotions, it should be considered as more controlled than gain framework information [75]. Therefore, we propose hypothesis H2:

**H2.**  *The information loss–gain framework promotes public PEB decision-making, and the effect of loss framework information is stronger than that of the gain framework.*

### 2.3. Coupling Information Intervention

Coupling information interventions (CIIs) are strategies that combine two or more different single information frameworks to promote public PEB. Coupling information interventions are thought to promote PEB in individuals better than single information campaigns or information feedback [16,76]. Delmas et al. suggested a 7.4% reduction in average individual electricity consumption by conducting a meta-analysis of 156 studies from 1975 to 2012 on energy-saving experiments based on information interventions [77]. Using meta-analysis techniques to synthesize 42 residential energy-saving experiments published between 1977 and 2014, Mi et al. found that information interventions had a positive effect on residential energy-saving behavior, but that different information frameworks resulted in significantly different energy savings, and that coupling interventions were more effective than single interventions [78]. Thondhlana and Kua [79], after using an experimental study of 103 households in South Africa, found that the combined intervention was more effective in promoting energy-savings than the individual interventions. Therefore, compared to the single information intervention effects of the information loss–gain and information content frameworks, the effectiveness of coupling information interventions of the two information frameworks is more worthy for exploring.

The PEB decision-making in this study involves multiple spheres, including the private sphere, work organizations, and public places. Unlike household users, the contribution of individuals, organizations and public places to energy-saving and environmental protection cannot be measured. Moreover, the benefits from energy-saving are shared externally. This results in a lack of strong motivation for people to actively implement PEB. Hence, the coupling information intervention of the information loss–gain framework and the information content framework seems to be more important. Therefore, the research question of this study is presented as whether coupling information interventions between the information loss–gain framework and the information content framework can help to promote individuals’ PEB choices. Which coupling intervention strategy is more effective? A 2 (an information gain framework and an information loss framework) × 2 (an information healthy framework vs. an information environmental framework) between-subjects experiment is set up to test the effectiveness of four intervention strategies, which couple an information loss–gain framework (loss framework vs. gain framework) with an information content framework (healthy information vs. environmental information) in promoting public PEB. Thus, we propose hypothesis H3:

**H3.**  *Interventions coupling the information loss–gain framework with the information content framework can significantly promote the public PEB decision-making, and there are significant differences in the effectiveness of the different coupling approaches.*

**H3a.**  *Environmental gain information can significantly contribute to the public PEB decision-making.*

**H3b.**  *Environmental loss information significantly promotes the public PEB decision-making.*

**H3c.**  *Healthy gain information significantly promotes the public PEB decision-making.*

**H3d.**  *Healthy loss information significantly promotes the public PEB decision-making.*

## 3. Method

### 3.1. Experimental Subjects

Due to the impact of the epidemic, offline experiments have been hampered, so our experiments were conducted online. Compared with offline experiments, the collection of samples for online experiments is not restricted by time and location, which makes it easier and faster, and reduces the waste caused by the inability to recollect in offline scenario.

Before the beginning of experiment, the sample size required for the experiment is calculated by G*Power 3.1, and we use a two-factor ANOVA. In G*Power 3.1, the two-factor ANOVA requires the setting of values for effect size, alpha, and test effectiveness power. The effect size (d) is divided into small (0.1), medium (0.25), and large (0.4) effects, where a larger effect size indicates a smaller overlap between the two aggregates and a more significant effect. To ensure the accuracy and reliability of the experiment, we set the effect size to 0.4 for the large effect. α is the confidence interval, generally set at 0.05, and the test power is generally set at the lower limit of 0.8. In this experiment, the four information intervention strategies formed by the coupling of the information loss–gain framework and the information content framework are the independent variables, so the number of groups is set to four, and the total sample size is greater than or equal to 280 to meet the statistical requirement [80]. Considering that this study divided PEB into three spheres: home sphere, workplace, and public sphere, a total of 320 volunteers were recruited for this study; the group of students with no work experience and the retired and non-working staff were not included. To increase the motivation of the participants, the staff informed them before the experiment started that they would be rewarded if they passed the audit. In the end, a total of 318 people participated in this experiment, and 292 valid samples were obtained after excluding the samples that took too short a time for the whole experiment. The sample size requirement was met. The sample structure is shown in Table 1.

### 3.2. Experimental Design

**Figure 1:**
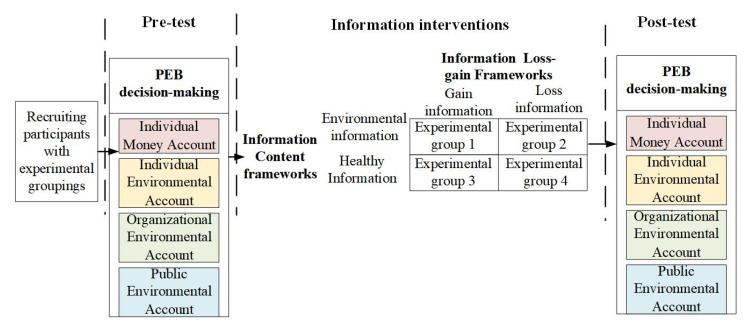
Experimental design diagram.

This experiment aimed at testing whether and how the COVID-19 pandemic information frameworks could facilitate public PEB decision-making more effectively. The experiment used the public PEB decision-making in different spheres, such as the private sphere, organizations, and public places, as the dependent variable. Additionally, the four information intervention strategies formed by coupling the information loss–gain framework with the information content framework are the independent variables. The experimental design is shown in Figure 1. A total of four experimental groups were designed to provide environmental gain information, environmental loss information, healthy gain information, and healthy loss information, respectively. Among them, environmental gain information refers to information about the positive outcome or positive effect of the COVID-19 epidemic event on the environment; environmental loss information refers to information about the loss or negative effect of the COVID-19 epidemic event on the environment; healthy gain information refers to information about the positive outcome or positive effect of the COVID-19 epidemic event on people’s health; healthy loss information refers to information about the loss or negative effect of the COVID-19 epidemic event on people’s health. The contents of the four information intervention strategies are shown in Table 2.

The measurement of public PEB decision-making is carried out through decision-making on the allocation of environmental credits. This is done by giving the public 100 initial points and informing them that they need to allocate all 100 points to four accounts: an individual money account, an individual environmental account, an organizational environmental account, and a public environmental account. The number of points in the individual environmental account represents the extent to which the participant is practicing PEB in the private sphere, and will be used for the purchase of environmental products by the individual (e.g., bus or metro card, eco-friendly shopping bag). The number of points in an organization’s environmental account represents the extent to which the participant has practiced PEB in the organization’s sphere, and will be donated to the individual’s business or organization to carry out environmental activities or purchase environmentally friendly products. The number of points in a public environmental account represents the extent to which the public practiced PEB in the public sphere, and will be donated to public welfare projects through the China online charity platform.

### 3.3. Experimental Procedure

To ensure the validity of the random grouping and a balanced sample across experimental subgroups, participants were assigned into an experimental group randomly by selecting their month of birth (January–March/April–June/July–September/October–December) upon entry into the experiment. The experimental process was divided into three phases.

In the first stage, the public PEB decision-making before the information intervention was tested. In this phase, participants were given an individual account with 100 initial points and told that they needed to allocate all 100 points to four different accounts according to their true intentions. The purpose of each account is shown in Table 3. After confirming understanding the function of each account, participants allocated the points. They could proceed to the next stage if, and only if, the sum of points allocated to the 4 accounts was 100.

In the second stage, each of the four groups of subjects were provided with the corresponding information framework intervention on the effects of COVID-19 pandemic. After the subjects had received the information intervention, changes in their PEB decision-making following the information intervention were tested. To enter this phase, participants in each of the four groups were first provided with four types of information materials: health gain information, healthy loss information, environmental gain information, and environmental loss information. Participants in each group were reminded of reading the information materials carefully. After reading, participants were awarded 100 points again and re-started the point allocation decision process, where these 100 points were allocated to four accounts: individual money account, individual environmental account, organizational environmental account, and public environmental account, and, as the same as the first stage, the sum of points in four accounts had to be 100 so that they could move on to the next stage.

In the third stage, participants completed the experiment by filling in their personal basic information and submitting it. Personal basic information, including gender, age, education level, number of family members, monthly household income, occupation, etc., was collected.

## 4. Experimental Data Analysis and Results

### 4.1. Analysis of Variance of Subjects’ PEB Decision-Making before the Experiment

To ensure the internal validity of the experiment, the validity of the random grouping of the experiment needed to be tested. Before the experiment, all participants were asked to allocate 100 initial points to the four accounts, and a test of between-group differences was conducted based on the allocation results. If the between-group differences between the four groups were not significant, the random grouping was valid.

The number of points allocated by participants to their individual money account, individual environmental account, organizational environmental account, and public environmental account represented their decision on whether to be environmental or not and in which area to invest in environmental protection. We tested the validity of the randomized grouping using a one-way ANOVA with the four information framework conditions as independent variables, and the results of the points were allocated to each account in the pre-test stage of the four groups as dependent variables. The results showed that there were no significant group differences in the allocation of points across accounts in the four experimental groups in the pre-test stage and that the randomized grouping was valid. This provided a good antecedent condition for the subsequent experimental intervention. Details of the results are shown in Table 4.

### 4.2. Analysis of the Effects of 4 Information Frameworks on the Public PEB Decision-Making

To examine the effect of four different COVID-19 pandemic event information frameworks on the intervention of the public PEB decision-making, this study conducted a paired-samples *t*-test using SPSS, and the results are shown in Table 5. Overall, all four information frameworks significantly promoted the public PEB decision-making to invest in environmental accounts and reduced the investment in individual money accounts. However, the effects of the four information frameworks differed significantly for different areas of environmental protection inputs. Thus, hypothesis H3 was valid.

In terms of inputs to individual environmental accounts, environmental gain information significantly contributed to the public PEB decision-making (t = −2.981, *p* = 0.004), whereas environmental loss information (t = −1.351, *p* = 0.182), healthy gain information (t = −0.857, *p* = 0.394), and healthy loss information (t = −0.159, *p* = 0.874) had no significant effect on PEB decision-making in the private sphere.

In terms of organizational environmental account inputs, environmental loss information (t = −2.091, *p* = 0.041) and healthy gain information (t = −3.304, *p* = 0.001) significantly contributed to public PEB decision-making. However, environmental gain information (t = −1.125, *p* = 0.265) and healthy loss information (t = −1.818, *p* = 0.073) did not have significant effect on both the public and private sphere.

In terms of public environmental account inputs, environmental gain information (t = −2.442, *p* = 0.017), environmental loss information (t = −4.908, *p* = 0.000), healthy gain information (t = −2.771, *p* = 0.007), and healthy loss information (t = −3.142, *p* = 0.002) all significantly contributed to the public’s pro-environmental behavioral input.

**Figure 2:**
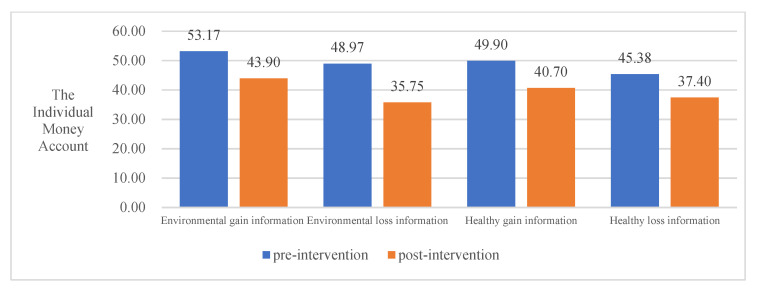
The impact of different information frameworks on the Individual Money Account.

**Figure 3:**
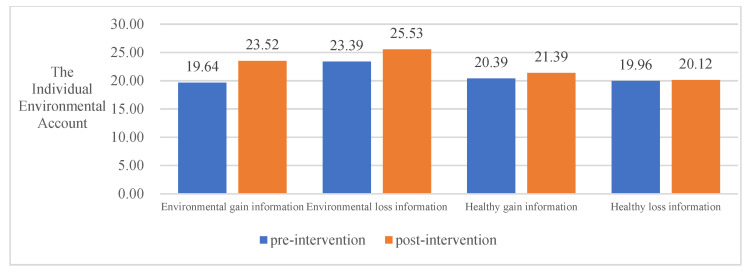
The impact of different information frameworks on the Individual Environmental Account.

**Figure 4:**
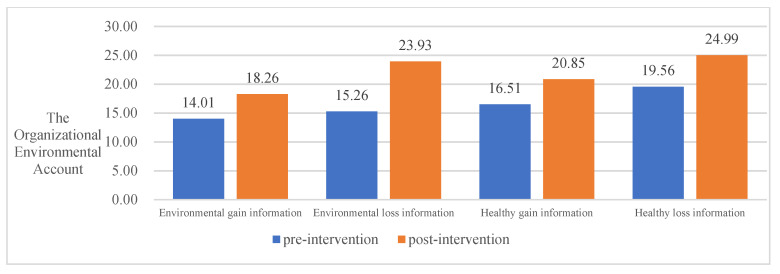
The impact of different information frameworks on the Organizational Environmental Account.

**Figure 5:**
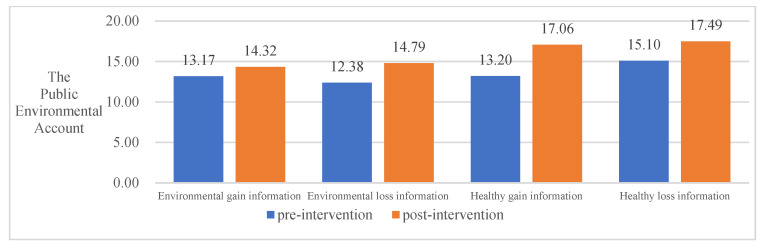
The impact of different information frameworks on the Public Environmental Account.

In summary, all four information frameworks significantly contribute to the public’s behavioral decision-making to invest in environmental accounts. Environmental loss information contributed most to the public PEB decision-making, followed by environmental gain information, healthy gain information, and healthy loss information. See Table 5 and Figure 2, Figure 3, Figure 4 and Figure 5 for details.

### 4.3. Factorial Analysis of the Information Content Framework and the Information Loss–Gain Framework

In the previous steps, we found that among the coupling interventions of the information loss–gain framework and the information content framework, the effects of all four coupling interventions on the public PEB decision-making were significant. To further test which information framework is more effective in intervening in the public PEB, we used a factorial analysis.

The first chi-squared test was conducted with the different information frameworks as the independent variables and the public PEB decision-making as the dependent variables. The results of the chi-squared test were F = 1.947, *p* = 0.122, which passed the test and allowed for the continuation of the univariate analysis of variance (ANOVA).

The purpose of the univariate ANOVA was to determine whether there was an interaction effect between the information loss–gain framework and the information content framework. The results of the univariate analysis of variance are shown in Table 6: the interaction between the loss–gain information framework and the content framework was not significant (F = 0.411, *p* = 0.522), so the effects of the two information frameworks on the public PEB decision-making were relatively independent, suggesting that a change in the level of one type of information did not affect the effect of the other. Therefore, we conducted a main effectiveness test.

The main effects analysis examines the extent to which a single factor affects the dependent variable. When the interaction effect is not significant, we can directly assess the magnitude of the effect of the independent variable on the dependent variable by checking whether its main effect is significant or not. The results of the main effects test are shown in Table 7. The effect of the information loss–gain framework on the public PEB decision-making is significant at the 10% level (F = 3.658, *p* = 0.057 < 0.1), and the effect of the information loss framework (M = 7.764, SD = 0.161) is greater than that of the gain framework (M = 7.334, SD = 0.157). Thus, hypothesis H2 is valid. The information content framework (F = 0.010, *p* = 0.922 > 0.1) had a non-significant effect on the public PEB decision-making, so hypothesis H1 is not valid.

## 5. Discussion

The objective of this experimental study was to investigate whether and how the framework effect of information on major PHEs can be used to promote PEB. Using the COVID-19 pandemic as a case, we bridged the gap between behavioral intentions and actual behavior. We designed the information about the COVID-19 pandemic into two information loss–gain frameworks (a gain framework and a loss framework) and two information content frameworks (environmental information and healthy information), and then coupled these two information frameworks to form four different information interventions. A pre- and post-test control experiment was conducted to measure changes in participants’ PEB decision-making when they were exposed to different information interventions. The results showed that all four information frameworks significantly promoted public PEB decision-making, but the effect of the different information frameworks on PEB decision-making in the three spheres differed significantly, which provided a new perspective on how to turn crises into opportunities to promote public PEB in the context of major PHEs.

First, even though all four coupling COVID-19 event information frameworks promoted public PEB decision-making, the effects of the different information frameworks differed significantly. Among them, environmental loss information had the greatest effect on promoting PEB decision-making by the public, followed by environmental gain information, healthy gain information, and healthy loss information plays the least role. This is similar to the findings of Ghesla et al. [72], an electricity-saving experiment on 1,636 households in a German region, which found that pro-environmental incentives combined with loss framework information saved 5% of electricity consumption per month compared to the control group. The study by Kahneman et al. found that, according to the Loss Aversion Theory, the emotional response that occurs when people are confronted with an immediate loss signal leads them to decide their inclination under conditions of uncertainty [61]. Driven by this thought, people will mostly respond more to losses when faced with equivalent gains and losses. Since the outbreak of COVID-19 pandemic in 2020, more than two years of prevention and control of the epidemic so far have made people nostalgic for the old days before the pandemic. The enormous changes and negative impacts on the environment and public life brought about by the COVID-19 pandemic are impressive. This is probably the most significant reason why information on environmental losses contributes to public PEB decision-making.

Secondly, there were significant differences in the effect of the different information frameworks in the three environmental spheres. Only the effect of environmental gain information was significant for PEB decision-making in the private sphere (t = −2.981, *p* = 0.004), whereas the effects of the other three information frameworks were not significant. One possibility is that PEB in the private sphere is an act of environmental protection that individuals voluntarily spend time and effort on [81]. The public can have a direct positive impact on the environment through PEB in the private sphere [82,83], resulting in the sense of contribution and environmental moral credibility [84]. Compared to other information, the public’s self-perception of the environmental gains arising from the implementation of PEB in the private sphere is more direct, and, thus, environmental gain information has a greater impact on the private sphere of public PEB decision-making.

For PEB decision-making in the organizational sphere, environmental loss information (t = −2.091, *p* = 0.041) and healthy gain information (t = −3.304, *p* = 0.001) significantly contributed to public PEB in this sphere, whereas the effects of environmental gain information and healthy loss information were not significant. This may be due to the long period of working from home caused by the epidemic. Moreover, the negative environmental information, as well as information about the impact on people’s health imposed by the COVID-19 pandemic, caused people to reflect on the situation, and they also realized the harm caused by environmental degradation and the importance of health. More people began to focus on maintaining a healthy lifestyle and improving their immunity. Therefore, the impact of environmental loss information and health gain information is greater.

In the public sphere, all four information frameworks significantly contribute to public PEB decision-making. In a time when epidemics are a regular occurrence, it is evident that the public is more willing to engage in PEB in the public sphere due to concerns about the epidemic. Furthermore, PEB in the public sphere is directly oriented toward the environment and sustainable development. The public’s influence on the public sphere may profoundly influence and change the behavior of others or organizations [82,83]. Therefore, the public is more inclined to discipline environmentally destructive behavior out of strong environmental claims during an epidemic. This shows that using the epidemic event as an opportunity to promote public PEB through event information management is a potential new path. Additionally, through different information frameworks, individuals can be targeted to promote the adoption of PEB in different areas.

Third, the factorial analysis found that the interaction effect of the information loss-gain framework and the information content framework on public PEB decision-making was not significant (F = 0.411, *p* = 0.522). The information loss–gain framework played a main role in the public PEB decision-making. In contrast, the effect of the information content framework was not significant and, of the effects of the information loss–gain framework on public behavioral decision-making, the loss framework outweighed the gain framework. This is similar to the results of previous studies [72,73]. According to prospect theory, the loss framework is more effective in changing risky behaviors and the gain framework is more effective in changing behaviors that are perceived as safe [85,86,87]. In general, when people encounter negative events, these events have a greater impact on them than positive events. Moreover, people usually process negative information more quickly and efficiently than positive information [88]. Some scholars have explained this difference from an evolutionary perspective, suggesting that ignoring negative signals is a greater threat to survival than ignoring positive signals [89]. This suggests that in the context of the COVID-19 pandemic, people are becoming increasingly aware of the dangers of environmental damage and the importance of environmental protection. The COVID-19 pandemic has brought about a 5% drop in global CO_2_ emissions [10,90], which has had a positive impact on the environment and a temporary gain. The COVID-19 pandemic has also caused widespread environmental pollution, with an estimated 129 billion masks and 65 billion gloves used globally each month [91]. In addition, personal protective equipment (PPE) and packaging materials are widely used to prevent the spread of COVID-19 pandemic, but are often poorly managed, generating large amounts of plastic waste [92]. Therefore, people are more willing to reduce the environmental or health hazards caused by COVID-19 by implementing environmental behaviors.

Fourth, inconsistent with our expectations, the factorial analysis found that neither single environmental information nor healthy information had a significant effect on public PEB decision-making. Previous studies have shown that information alone has little influence on long-term energy conservation behavior and the presentation of behavioral outcomes [47,77]. Steinhorst and Klöckner’s [93] study demonstrate that a single environmental information framework does not affect long-term PEBchange. Geng et al. also found in an experimental study of non-motorized travel by vehicle owners that providing environmental information had no significant effect on encouraging non-motorized travel by vehicle owners, but that combining it with health information promoted increased walking and cycling time in the short term [94]. Thus, as the COVID-19 pandemic spreads and persists globally, single healthy information and single environmental information about environmental protection can no longer be strong predictors of people’s implementation of PEB. In contrast, individuals’ PEB is more likely to be influenced by a sense of norm and responsibility [95]. In conjunction with Horng and Heidbreder [95,96], a possible reason for the research on the role of information in promoting environmental behavior is that single environmental information and single health information can stimulate public awareness of environmental protection, as well as increase public perceptions of PEB. However, it is difficult to turn them into specific PEB decision-making. Another reason is that the perceived consequences of a single piece of environmental or health information are not strong. It takes a combination of environmental and health information to get enough attention from participants and to pay for changes in behavioral decision-making.

## 6. Conclusions

Through an online pre-and post-test control experiment, this study investigated the effects of four information frameworks effects of different PHE information on the public PEB decision-making, using the COVID-19 pandemic as a case. The study coupled the information loss–gain and information content frameworks to form four information intervention strategies, as well as measured and analyzed the effects of PEB decision-making in the form of environmental credit allocation. The results found that all four coupling information frameworks significantly contributed to the implementation of public PEB. However, there were significant differences in the effects of different information frameworks on individual PEB decision-making in the private, organizational, and public spheres. Further factorial analysis reveals that there is no interaction between the information content framework and the information loss–gain framework. The information content framework does not contribute significantly to changes in PEB decision-making. The information loss–gain framework plays the dominant role, and the loss framework is significantly more effective than the gain framework.

In the practical realm, this paper provides some new insights into how information frameworks can be used to motivate the public to make PEB decision-making in the context of major PHEs. First, in the context of major PHEs, government departments and environmental management agencies need to make use of the framework effect of event information in environmental information dissemination, especially in the environmental loss information related to PHEs as the focus of information design for playing an active role in promoting public PEB decision-making. Second, in organizational units, in the context of major PHEs, environmental loss information and healthy gain information needs to be highlighted to promote public PEB in organizations. Thirdly, for the private sphere, such as households, policymakers should strongly advocate environmental gain information associated with major PHEs to stimulate PEB in the private sphere.

Although this study yielded some positive and valuable findings, there are still some limitations: (1) this study focused on the effects of the information loss–gain framework and the information content framework in the design of information on the COVID-19 pandemic events. However, in the Internet era, the impact of different forms of information (e.g., text, pictures, videos) on public behavior is also a point of concern. Therefore, different forms of information can be further designed in future studies to test which information form has a better effect on individual PEB and enrich the effect of information intervention. (2) Due to the limitation of the sample size, we only examined the intervention strategies of coupling the information content framework and the information loss–gain framework on public PEB decision-making and did not consider the effect of a single piece of information. The effects of different information strategies on PEB can be further explored in future studies. (3) Emotions have also been shown to influence public PEB. The use of emotions as a mediating variable to investigate the relationship between information interventions and environmental behavior are also worth for further investigation. (4) This study used quantitative data to assess the effects of the experiment and did not collect qualitative data through open-ended questions. In the future, qualitative data such as respondents’ views can be obtained through in-depth interviews to better analyze the psychological cognitive processes behind the behavioral outcomes. In addition, future research could further explore the influence of sociodemographic characteristics on PEB decision-making under different information frameworks if the sample size is large enough.

## Figures and Tables

**Table 1:** Participant’s Descriptive Statistical Analysis Results.

Variable	Category	N	Percentage	Variable	Category	N	Percentage
Gender	Male	143	49%	Education level	Below junior high school	5	1.7%
	Female	149	51%		Senior high school or secondary school degree	17	5.8%
Monthly household income	<3000 RMB	28	9.6%		Bachelor degree	155	53.1%
	3000–5000 RMB	46	15.8%		Graduate degree	115	39.4%
	5000–10,000 RMB	73	25.0%	Age	<20	2	0.7%
	10,000–20,000 RMB	98	33.6%		20–30	122	41.8%
	20,000–50,000 RMB	34	11.6%		31–40	81	27.7%
	>50,000 RMB	13	4.4%		41–50	56	19.2%
Occupation	Engineer	51	17.5%		>50	31	10.6%
	Staff working in non-profit organizations such as scientific research, education, medical care and other fields	79	27.1%	Number of family members	1	12	4.1%
	Enterprise manager	55	18.8%		2	26	8.9%
	General workers or service personnel	14	4.8%		3	119	40.8%
	Government staff	17	5.8%		4	80	27.4%
	Freelancers	14	4.8%		>5	55	18.8%
	Others	62	21.2%				

**Table 2:** The contents of the four information intervention strategies.

Name	Information Intervention Specifics
Environmental gain information	From January to November 2022, the proportion of good air days in China’s 339 cities was 86.8%, up 4.8 percentage points from 2019; in 2021, Beijing had an average of 5.5 good PM2.5 days out of 7 days per week, compared to 4.6 days in 2019. Under the regular epidemic prevention and control measures, air quality has been significantly improved as a result.
Environmental loss information	Data released by the Ministry of Ecology and Environment of the People’s Republic of China shows that in 2021, a total of 1.4 million tons of medical waste will be generated nationwide, of which 211,000 tons will be involved in epidemics, an increase of 18.6% and 11.1% over 2019 and 2020, respectively. If we do not learn from the lessons of the epidemic and protect the environment while fighting it, a new crisis of ecological degradation or environmental pollution will come.
Health gain information	Data from the Chinese Centre for Disease Control and Prevention shows that the number of influenza cases in China from 1.14 million in 2020 to 420,000 in 2021. In the context of the COVID-19 pandemic, the concept of wearing masks and washing hands regularly to prevent epidemics has been significantly strengthened.
Health loss information	WHO data showed that as of 31 March 2022 Beijing time, the COVID-19 pandemic has caused 486,149,869 confirmed cases and 6,160,787 deaths worldwide. This shows that the health threat posed to us by the COVID-19 pandemic is enormous.

**Table 3:** Definitions of the four types of accounts.

Account Name	Account Definition
Individual Money Account	The points allocated to this account are at the disposal of the individual, but not for environmental expenditure
Individual Environmental Account	The points allocated to this account will be used for personal purchases of environmental products (e.g., bus or metro cards, environmental shopping bags)
Organizational Environmental Account	The points allocated to this account will be donated to companies or organizations to carry out environmental activities or purchase environmental products
Public Environmental Account	The points allocated to this account will be donated to charity projects through the Alipay platform (building wildlife sanctuaries, launching blue sky campaigns, etc.)

**Table 4:** Between-group difference test for initial point allocation for participants in the four pre-test groups.

Dependent Variable	Independent Variable	N	Mean	SD	F-Test Value	*p*-Value
Individual Money Account	Environmental gain information	69	53.17	24.651	1.047	0.372
	Environmental loss information	61	48.97	27.194		
	Healthy gain information	80	49.90	28.531		
	Healthy loss information	82	45.38	28.007		
	Total	292	49.21	27.236		
Individual Environmental Account	Environmental gain information	69	19.64	13.605	0.983	0.401
	Environmental loss information	61	23.39	16.022		
	Healthy gain information	80	20.39	13.301		
	Healthy loss information	82	19.96	13.114		
	Total	292	20.72	13.930		
Organizational Environmental Account	Environmental gain information	69	13.17	11.061	0.791	0.500
	Environmental loss information	61	12.38	10.162		
	Healthy gain information	80	13.20	11.671		
	Healthy loss information	82	15.10	11.720		
	Total	292	13.55	11.231		
Public Environmental Account	Environmental gain information	69	14.01	15.120	1.569	0.197
	Environmental loss information	61	15.26	14.699		
	Healthy gain information	80	16.51	15.474		
	Healthy loss information	82	19.56	19.675		
	Total	292	16.52	16.587		

**Table 5:** Table of paired sample *t*-test results of different information frameworks on different spheres of the public.

Dependent Variable	Information Interventions	N	Mean	SD	Uniform Deviation	t	*p*
Individual Money Account	Environmental gain information (pre-intervention)	69	53.17	16.697	−9.275	4.614	0.000
	Environmental gain information (post-intervention)	69	43.90				
	Environmental loss information (pre-intervention)	61	48.97	15.840	−13.313	6.515	0.000
	Environmental loss information (post-intervention)	61	35.75				
	Healthy gain information (pre-intervention)	80	49.90	12.498	−9.2000	6.584	0.000
	Healthy gain information (post-intervention)	80	40.70				
	Healthy loss information (pre-intervention)	82	45.38	13.214	−7.976	5.465	0.000
	Healthy loss information (post-intervention)	82	37.40				
Individual Environmental Account	Environmental gain information (pre-intervention)	69	19.64	10.824	3.884	−2.981	0.004
	Environmental gain information (post-intervention)	69	23.52				
	Environmental loss information (pre-intervention)	61	23.39	12.317	2.131	−1.351	0.182
	Environmental loss information (post-intervention)	61	25.53				
	Healthy gain information (pre-intervention)	80	20.39	10.439	1.000	−0.857	0.394
	Healthy gain information (post-intervention)	80	21.39				
	Healthy loss information (pre-intervention)	82	19.96	9.048	0.159	−0.159	0.874
	Healthy loss information (post-intervention)	82	20.12				
Organizational Environmental Account	Environmental gain information (pre-intervention)	69	13.17	8.456	1.145	−1.125	0.265
	Environmental gain information (post-intervention)	69	14.32				
	Environmental loss information (pre-intervention)	61	12.38	9.002	2.401	−2.091	0.041
	Environmental loss information (post-intervention)	61	14.79				
	Healthy gain information (pre-intervention)	80	13.20	10.458	3.863	−3.304	0.001
	Healthy gain information (post-intervention)	80	17.06				
	Healthy loss information (pre-intervention)	82	15.10	11.904	2.390	−1.818	0.073
	Healthy loss information (post-intervention)	82	17.49				
Public Environmental Account	Environmental gain information (pre-intervention)	69	14.01	14.446	4.246	−2.442	0.017
	Environmental gain information (post-intervention)	69	18.26				
	Environmental loss information (pre-intervention)	61	15.26	13.801	8.672	−4.908	0.000
	Environmental loss information (post-intervention)	61	23.93				
	Healthy gain information (pre-intervention)	80	16.51	13.999	4.338	−2.771	0.007
	Healthy gain information (post-intervention)	80	20.85				
	Healthy loss information (pre-intervention)	82	19.56	15.639	5.427	−3.142	0.002
	Healthy loss information (post-intervention)	82	24.99				

**Table 6:** Inter-subject effects test.

Source	Class III Sum of Squares	df	Mean Square	F-Value	Sig.	Partial Eta Square
Modified model	13.984 ^a^	3	4.661	1.281	0.281	0.013
Intercept distance	16,405.382	1	16,405.382	4509.397	0.000	0.940
Information loss–gain framework	13.307	1	13.307	3.658	0.057	0.013
Information content framework	0.035	1	0.035	0.010	0.922	0.000
Information loss–gain × Information content	1.497	1	1.497	0.411	0.522	0.001
Error	1047.756	288	3.638			
Total	17,667.000	292				
Total after amendment	1061.740	291				

^a^ R-square = 0.013 (adjusted R-square = 0.003). Note: according to Cohen (1988), partial eta square >= 0.14 represents a large effect, 0.14 > partial eta square >= 0.06 a medium effect, and 0.06 > partial eta square >= 0.01 a small effect.

**Table 7:** Main effects test.

Independent Variable 1	Independent Variable 2	F	Sig.	Partial Eta Square	Mean	SD	95% Confidence Interval	
							Low	High
Information loss-gain framework	Gain framework	3.658	0.057	0.013	7.334	0.157	−0.873	0.013
	Loss framework				7.764	0.161	0.013	−0.873
Information content framework	Environmental information	0.010	0.922	0.000	7.560	0.168	−0.420	0.465
	Healthy Information				7.538	0.150	0.465	−0.420

## Data Availability

The data presented in this study are available within the article.

## References

[1] Tilman D., Clark M. (2014). Global diets link environmental sustainability and human health. Nature.

[2] Severo E.A., De Guimarães J.C.F., Dellarmelin M.L. (2021). Impact of the COVID-19 pandemic on environmental awareness, sustainable consumption and social responsibility: Evidence from generations in Brazil and Portugal. J. Clean. Prod..

[3] Cuevas-Ferrando E., Girón-Guzmán I., Falcó I., Pérez-Cataluña A., Díaz-Reolid A., Aznar R., Randazzo W., Sánchez G. (2022). Discrimination of non-infectious SARS-CoV-2 particles from fomites by viability RT-qPCR. Environ. Res..

[4] Zhao X.X., Wen J., Zou X.Y., Wang Q.J., Chang C.P. (2022). Strategies for the sustainable development of China in the post-epidemic era. Sustain. Dev..

[5] Mostafa M.K., Gamal G., Wafiq A. (2021). The impact of COVID 19 on air pollution levels and other environmental indicators—A case study of Egypt. J. Environ. Manag..

[6] Zeng J., Bao R. (2021). The impacts of human migration and city lockdowns on specific air pollutants during the COVID-19 outbreak: A spatial perspective. J. Environ. Manag..

[7] Dias Á.L., Silva R., Patuleia M., Estêvão J., González-Rodríguez M.R. (2022). Selecting lifestyle entrepreneurship recovery strategies: A response to the COVID-19 pandemic. Tour. Hosp. Res..

[8] Gong Y., Zhang L., Sun Y. (2021). Correction: More than just a mental stressor: Psychological value of social distancing in COVID-19 mitigation through increased risk perception—A preliminary study in China. Humanit. Soc. Sci. Commun..

[9] Chakraborty I., Maity P. (2020). COVID-19 outbreak: Migration, effects on society, global environment and prevention. Sci. Total Environ..

[10] Tchetchik A., Kaplan S., Blass V. (2021). Recycling and consumption reduction following the COVID-19 lockdown: The effect of threat and coping appraisal, past behavior and information. Resour. Conserv. Recycl..

[11] Silva A.C.T., Branco P.T., Ferrini Rodrigues P., Sousa S.I. (2022). Sustainable policies for air pollution reduction after COVID-19 pandemic: Lessons learnt from the impact of the different lockdown periods on air quality. Sustain. Dev..

[12] Leal Filho W., Salvia A.L., Paço A., Dinis M.A.P., Vidal D.G., Da Cunha D.A., de Vasconcelos C.R., Baumgartner R.J., Rampasso I., Anholon R. (2022). The influences of the COVID-19 pandemic on sustainable consumption: An international study. Environ. Sci. Eur..

[13] Kanda W., Kivimaa P. (2020). What opportunities could the COVID-19 outbreak offer for sustainability transitions research on electricity and mobility?. Energy Res. Soc. Sci..

[14] Muhammad S., Long X., Salman M. (2020). COVID-19 pandemic and environmental pollution: A blessing in disguise?. Sci. Total Environ..

[15] Sarkis J., Cohen M.J., Dewick P., Schröder P. (2020). A brave new world: Lessons from the COVID-19 pandemic for transitioning to sustainable supply and production. Resour. Conserv. Recycl..

[16] Mi L., Zhao J., Xu T., Yang H., Lv T., Shang K., Qiao Y., Zhang Z. (2021). How does COVID-19 emergency cognition influence public pro-environmental behavioral intentions? An affective event perspective. Resour. Conserv. Recycl..

[17] Shulman D., Halperin E., Reifen-Tagar M. (2022). Personal experience with COVID-19 is associated with increased environmental concern and pro-environmental behavioral intentions. Curr. Res. Ecol. Soc. Psychol..

[18] O’Connor P., Assaker G. (2022). COVID-19’s effects on future pro-environmental traveler behavior: An empirical examination using norm activation, economic sacrifices, and risk perception theories. J. Sustain. Tour..

[19] Sun Q., Willemsen M.C., Knijnenburg B.P. (2020). Unpacking the intention-behavior gap in privacy decision making for the internet of things (IoT) using aspect listing. Comput. Secur..

[20] Grimmer M., Miles M.P. (2017). With the best of intentions: A large sample test of the intention-behaviour gap in pro-environmental consumer behaviour: Intention-behaviour gap in PECB. Int. J. Consum. Stud..

[21] Sheng G., Dai J., Pan H. (2020). Influence of Air Quality on Pro-environmental Behavior of Chinese Residents: From the Perspective of Spatial Distance. Front. Psychol..

[22] Blok V., Wesselink R., Studynka O., Kemp R. (2015). Encouraging sustainability in the workplace: A survey on the pro-environmental behaviour of university employees. J. Clean. Prod..

[23] Wyss A.M., Knoch D., Berger S. (2022). When and how pro-environmental attitudes turn into behavior: The role of costs, benefits, and self-control. J. Environ. Psychol..

[24] Meng L., Si W. (2022). Pro-Environmental Behavior: Examining the Role of Ecological Value Cognition, Environmental Attitude, and Place Attachment among Rural Farmers in China. Int. J. Environ. Res. Public Health.

[25] Mi L., Qiao L., Xu T., Gan X., Yang H., Zhao J., Qiao Y., Hou J. (2020). Promoting sustainable development: The impact of differences in cultural values on residents’ pro-environmental behaviors. Sustain. Dev..

[26] Ahmat Zainuri N., Abd-Rahman N., Halim L., Chan M.Y., Mohd Bazari N.N. (2022). Measuring Pro-Environmental Behavior Triggered by Environmental Values. Int. J. Environ. Res. Public Health.

[27] Scopelliti M., Barni D., Rinallo E. (2022). My Parents Taught… Green Was My Growth! The Role of Intergenerational Transmission of Ecological Values in Young Adults’ Pro-Environmental Behaviors and Their Psychosocial Mechanisms. Int. J. Environ. Res. Public Health.

[28] Ru X., Wang S., Yan S. (2018). Exploring the effects of normative factors and perceived behavioral control on individual’s energy-saving intention: An empirical study in eastern China. Resour. Conserv. Recycl..

[29] González-Rodríguez M.R., Tussyadiah I. (2022). Sustainable development in nature-based destinations. The social dilemma of an environmental policy. Sustain. Dev..

[30] Zhang Y., Wang Z., Zhou G. (2014). Determinants of employee electricity saving: The role of social benefits, personal benefits and organizational electricity saving climate. J. Clean. Prod..

[31] Nolan J.M., Schultz P.W., Cialdini R.B., Goldstein N.J., Griskevicius V. (2008). Normative social influence is underdetected. Personal. Soc. Psychol. Bull..

[32] Sun Y., Li P., She S., Eimontaite I., Yang B. (2018). Boosting water conservation by improving campaign: Evidence from a field study in China. Urban Water J..

[33] Xiao S., Dong H., Geng Y., Tian X., Liu C., Li H. (2020). Policy impacts on Municipal Solid Waste management in Shanghai: A system dynamics model analysis. J. Clean. Prod..

[34] Xu L., Ling M., Lu Y., Shen M. (2017). External influences on forming residents’ waste separation behaviour: Evidence from households in Hangzhou, China. Habitat Int..

[35] Brécard D., Hlaimi B., Lucas S., Perraudeau Y., Salladarré F. (2009). Determinants of demand for green products: An application to eco-label demand for fish in Europe. Ecol. Econ..

[36] Botetzagias I., Dima A.F., Malesios C. (2015). Extending the theory of planned behavior in the context of recycling: The role of moral norms and of demographic predictors. Resour. Conserv. Recycl..

[37] Paço A., Lavrador T. (2017). Environmental knowledge and attitudes and behaviours towards energy consumption. J. Environ. Manag..

[38] Salancik G.R., Pfeffer J. (1978). A social information processing approach to job attitudes and task design. Adm. Sci. Q..

[39] Zebardast L., Radaei M. (2022). The influence of global crises on reshaping pro-environmental behavior, case study: The COVID-19 pandemic. Sci. Total Environ..

[40] Broomell S.B., Chapman G.B. (2021). Looking Beyond Cognition for Risky Decision Making: COVID-19, the Environment, and Behavior. J. Appl. Res. Mem. Cogn..

[41] Viksnin I.I., Gataullin R., Muradov A., Danilov I., Tursukov N., Chechet A. Modeling people behavior in emergency situations. Proceedings of the 2017 20th Conference of Open Innovations Association (FRUCT).

[42] Levin I.P., Schneider S.L., Gaeth G.J. (1998). All Frames Are Not Created Equal: A Typology and Critical Analysis of Framing Effects. Organ. Behav. Hum. Decis. Process..

[43] Mollen S., Holland R.W., Ruiter RA C., Rimal R.N., Kok G. (2021). When the Frame Fits the Social Picture: The Effects of Framed Social Norm Messages on Healthy and Unhealthy Food Consumption. Commun. Res..

[44] Gallagher K.M., Updegraff J.A. (2012). Health Message Framing Effects on Attitudes, Intentions, and Behavior: A Meta-analytic Review. Ann. Behav. Med..

[45] Han X., Deng J., Han W. (2022). The mechanism of information framework’s influence on residents’ initial vaccination intention of new crown vaccine—A moderated mediation model. Mod. Intell..

[46] Scopelliti M., Pacilli M.G., Aquino A. (2021). TV news and COVID-19: Media influence on healthy behavior in public spaces. Int. J. Environ. Res. Public Health.

[47] Abrahamse W., Steg L., Vlek C., Rothengatter T. (2005). A review of intervention studies aimed at household energy conservation. J. Environ. Psychol..

[48] Mi L., Qiao L., Gan X., Xu T., Lv T., Qiao Y., Ding C. (2020). Assessing the effect of non-financial information intervention on promoting group-level energy savings. Sci. Total Environ..

[49] Kamilaris A., Neovino J., Kondepudi S., Kalluri B. (2015). A case study on the individual energy use of personal computers in an office setting and assessment of various feedback types toward energy savings. Energy Build..

[50] Asensio O.I., Delmas M.A. (2015). Nonprice incentives and energy conservation. Proc. Natl. Acad. Sci. USA.

[51] Asensio O.I., Delmas M.A. (2016). The dynamics of behavior change: Evidence from energy conservation. J. Econ. Behav. Organ..

[52] Chen V.L., Delmas M.A., Locke S.L., Singh A. (2017). Information strategies for energy conservation: A field experiment in India. Energy Econ..

[53] Mi L., Qiao L., Du S., Xu T., Gan X., Wang W., Yu X. (2020). Evaluating the effect of eight customized information strategies on urban households’ electricity saving: A field experiment in China. Sustain. Cities Soc..

[54] Corral-Verdugo V., Corral-Frías N.S., Frías-Armenta M., Lucas M.Y., Peña-Torres E.F. (2021). Positive Environments and Precautionary Behaviors During the COVID-19 Outbreak. Front. Psychol..

[55] Si H., Shen L., Liu W., Wu G. (2021). Uncovering people’s mask-saving intentions and behaviors in the post-COVID-19 period: Evidence from China. Sustain. Cities Soc..

[56] Brandon G., Lewis A. (1999). Reducing household energy consumption: A qualitative and quantitative field study. J. Environ. Psychol..

[57] Castellari E., Ricci E.C., Stranieri S., Marette S., Sarnataro M., Soregaroli C. (2019). Relationships between health and environmental information on the willingness to pay for functional foods: The case of a new aloe vera based product. Nutrients.

[58] Myers T.A., Nisbet M.C., Maibach E.W., Leiserowitz A.A. (2012). A public health frame arouses hopeful emotions about climate change. Clim. Chang..

[59] Wolske K.S., Todd A., Rossol M., McCall J., Sigrin B. (2018). Accelerating demand for residential solar photovoltaics: Can simple framing strategies increase consumer interest?. Glob. Environ. Chang..

[60] Bimonte S., Bosco L., Stabile A. (2020). Nudging pro-environmental behavior: Evidence from a web experiment on priming and WTP. J. Environ. Plan. Manag..

[61] Kahneman D., Tversky A. (1979). Prospect Theory: An Analysis of Decision under Risk. Econometrica.

[62] Tversky A., Kahneman D. (1985). The framing of decisions and the psychology of choice. Behavioral Decision Making.

[63] Mays D., Evans W.D. (2017). The Effects of Gain-, Loss-, and Balanced-Framed Messages for Preventing Indoor Tanning among Young Adult Women. J. Health Commun..

[64] Lee H., Cameron G.T. (2017). Utilizing Audiovisual and Gain-Framed Messages to Attenuate Psychological Reactance Toward Weight Management Health Messages. Health Commun..

[65] Moser S.C. (2010). Communicating climate change: History, challenges, process and future directions. WIREs Clim. Chang..

[66] Nabi R.L., Walter N., Oshidary N., Endacott C.G., Love-Nichols J., Lew Z.J., Aune A. (2020). Can Emotions Capture the Elusive Gain-Loss Framing Effect? A Meta-Analysis. Commun. Res..

[67] Mays D., Zhao X. (2016). The influence of framed messages and self-affirmation on indoor tanning behavioral intentions in 18- to 30-year-old women. Health Psychol..

[68] Ort A., Reinhardt A., Koch L., Rossmann C. (2021). The Emotional Effects of Gain-Loss Frames in Persuasive Messages about Sun Protection on Health Promotional Outcomes: Evidence from an Experimental Study. Health Commun..

[69] Iwasaki S., Franssens S., Dewitte S., Lange F. (2021). Evaluating the Effect of Framing Energy Consumption in Terms of Losses versus Gains on Air-Conditioner Use: A Field Experiment in a Student Dormitory in Japan. Sustainability.

[70] Dai S., Chen K., Jin R. (2022). The effect of message framing and language intensity on green consumption behavior willingness. Environ. Dev. Sustain..

[71] Bager S., Mundaca L. (2017). Making ‘Smart Meters’ smarter? Insights from a behavioural economics pilot field experiment in Copenhagen, Denmark. Energy Res. Soc. Sci..

[72] Ghesla C., Grieder M., Schmitz J., Stadelmann M. (2020). Pro-environmental incentives and loss aversion: A field experiment on electricity saving behavior. Energy Policy.

[73] Ropret Homar A., Knežević Cvelbar L. (2021). The effects of framing on environmental decisions: A systematic literature review. Ecol. Econ..

[74] Kim S.-B., Kim D.-Y. (2014). The Effects of Message Framing and Source Credibility on Green Messages in Hotels. Cornell Hosp. Q..

[75] Witte K. (1992). Putting the fear back into fear appeals: The extended parallel process model. Commun. Monogr..

[76] Karlin B., Zinger J.F., Ford R. (2015). The effects of feedback on energy conservation: A meta-analysis. Psychol. Bull..

[77] Delmas M.A., Fischlein M., Asensio O.I. (2013). Information strategies and energy conservation behavior: A meta-analysis of experimental studies from 1975 to 2012. Energy Policy.

[78] Mi L., Yang J., Yu X., Du L. (2016). Research on the intervention effect of information-based strategy on residents’ energy-saving behavior—Based on Meta-analysis. Soft Sci..

[79] Thondhlana G., Kua H.W. (2016). Promoting household energy conservation in low-income households through tailored interventions in Grahamstown, South Africa. J. Clean. Prod..

[80] Faul F., Erdfelder E., Lang A.-G., Buchner A. (2007). G*Power 3: A flexible statistical power analysis program for the social, behavioral, and biomedical sciences. Behav. Res. Methods.

[81] Rice G. (2006). Pro-environmental Behavior in Egypt: Is there a Role for Islamic Environmental Ethics?. J. Bus. Ethics.

[82] Liobikienė G., Poškus M.S. (2019). The Importance of Environmental Knowledge for Private and Public Sphere Pro-Environmental Behavior: Modifying the Value-Belief-Norm Theory. Sustainability.

[83] Stern P.C. (2000). New Environmental Theories: Toward a Coherent Theory of Environmentally Significant Behavior. J. Soc. Issues.

[84] Liao Y., Yang W. (2022). The determinants of different types of private-sphere pro-environmental behaviour: An integrating framework. Environ. Dev. Sustain..

[85] Banks S.M., Salovey P., Greener S., Rothman A.J., Moyer A., Beauvais J., Epel E. (1995). The effects of message framing on mammography utilization. Health Psychol..

[86] Spence A., Pidgeon N. (2010). Framing and communicating climate change: The effects of distance and outcome frame manipulations. Glob. Environ. Chang..

[87] O’Keefe D.J., Jensen J.D. (2009). The Relative Persuasiveness of Gain-Framed and Loss-Framed Messages for Encouraging Disease Detection Behaviors: A Meta-Analytic Review. J. Commun..

[88] Bracken C.C., Jeffres L.W., Neuendorf K.A. (2004). Criticism or praise? The impact of verbal versus text-only computer feedback on social presence, intrinsic motivation, and recall. Cyberpsychol. Behav..

[89] Bandura A. (1989). Human agency in social cognitive theory. Am. Psychol..

[90] Le Quéré C., Peters G.P., Friedlingstein P., Andrew R.M., Canadell J.G., Davis S.J., Jackson R.B., Jones M.W. (2021). Fossil CO_2_ emissions in the post-COVID-19 era. Nat. Clim. Chang..

[91] Prata J.C., Silva A.L., Walker T.R., Duarte A.C., Rocha-Santos T. (2020). COVID-19 Pandemic Repercussions on the Use and Management of Plastics. Environ. Sci. Technol..

[92] Igalavithana A.D., Yuan X., Attanayake C.P., Wang S., You S., Tsang D.C., Nzihou A., Ok Y.S. (2022). Sustainable management of plastic wastes in COVID-19 pandemic: The biochar solution. Environ. Res..

[93] Steinhorst J., Klöckner C.A. (2018). Effects of Monetary Versus Environmental Information Framing: Implications for Long-Term Pro-Environmental Behavior and Intrinsic Motivation. Environ. Behav..

[94] Geng J., Long R., Yang L., Zhu J., Engeda Birhane G. (2020). Experimental Evaluation of Information Interventions to Encourage Non-Motorized Travel: A Case Study in Hefei, China. Sustainability.

[95] Horng J.-S., Hu M.-L.M., Teng C.-C.C., Lin L. (2014). Energy Saving and Carbon Reduction Behaviors in Tourism—A Perception Study of Asian Visitors from a Protection Motivation Theory Perspective. Asia Pac. J. Tour. Res..

[96] Heidbreder L.M., Bablok I., Drews S., Menzel C. (2019). Tackling the plastic problem: A review on perceptions, behaviors, and interventions. Sci. Total Environ..

